# Calorie Anticipation Alters Food Intake After Low-Caloric but Not High-Caloric Preloads

**DOI:** 10.1002/oby.20293

**Published:** 2013-01-02

**Authors:** PS Hogenkamp, J Cedernaes, CD Chapman, H Vogel, OC Hjorth, S Zarei, LS Lundberg, SJ Brooks, SL Dickson, C Benedict, HB Schiöth

**Affiliations:** 1 Department of Neuroscience, Uppsala UniversitySE-751 24 Uppsala, Sweden; 2 Department of Physiology/Endocrinology, The Sahlgrenska Academy at the University of Gothenburg405 30 Gothenburg, Sweden

## Abstract

**Objective:**

Cognitive factors and anticipation are known to influence food intake. The current study examined the effect of anticipation and actual consumption of food on hormone (ghrelin, cortisol, and insulin) and glucose levels, appetite and *ad libitum* intake, to assess whether changes in hormone levels might explain the predicted differences in subsequent food intake.

**Design and Methods:**

During four breakfast sessions, participants consumed a yogurt preload that was either low caloric (LC: 180 kcal/300 g) or high caloric (HC: 530 kcal/300 g) and was provided with either consistent or inconsistent calorie information (i.e., stating the caloric content of the preload was low or high). Appetite ratings and hormone and glucose levels were measured at baseline (*t* = 0), after providing the calorie information about the preload (*t* = 20), after consumption of the preload (*t* = 40), and just before *ad libitum* intake (*t* = 60).

**Results:**

*Ad libitum* intake was lower after HC preloads (as compared to LC preloads; *P* < 0.01). Intake after LC preloads was higher when provided with (consistent) LC information (467±254 kcal) as compared to (inconsistent) HC information (346±210 kcal), but intake after the HC preloads did not depend on the information provided (LC information: 290±178 kcal, HC information: 333±179 kcal; caloric load*information *P* = 0.03). Hormone levels did not respond in an anticipatory manner, and the post-prandial responses depended on actual calories consumed.

**Conclusions:**

These results suggest that both cognitive and physiological information determine food intake. When actual caloric intake was sufficient to produce physiological satiety, cognitive factors played no role; however, when physiological satiety was limited, cognitively induced satiety reduced intake to comparable levels.

## Introduction

Cognitive factors are known to influence short-term food intake in humans (1,2): food anticipation as well as expectations regarding energy content, macronutrient content, or health aspects of food have been shown to influence intake (3–6), taste perception and preference (7,8), and expected and perceived satiety (9). Moreover, food anticipation affects physiological responses involved in the control of food intake. For example, external cues, such as food pictures (10) or labels providing “indulgent” information (11), elevate levels of the orexigenic hormone ghrelin. Ghrelin plays an important role in the short-term control of food intake: it stimulates meal initiation and produces a quick and robust increase in consumption (12,13). Ghrelin concentrations rise in humans during meal anticipation (14) and decrease in proportion to the amount of calories consumed (15,16). In a recent study, postprandial suppression of ghrelin levels was stronger in individuals who anticipated food intake as compared to those who did not expect a meal (17). Also, HPA-axis activity may be affected by food anticipation (18), therewith possibly modulating cortisol levels that are observed after food intake (19–21). However, it is not clear whether cognitive cues modulate (postprandial) ghrelin responses when the anticipated foods differ in their energy content and whether these changes in hormone levels may contribute to possible differences in subsequent food intake. To test this, participants in the present study were offered two low-caloric (LC) and two high-caloric (HC) preloads over four visits. Both the LC and HC preloads were presented once with consistent calorie information (i.e., low or high) and once with inconsistent calorie information (i.e., high or low). Weassessed pre-prandial and post-prandial plasma levels of ghrelin, as well as cortisol concentrations. In addition, LC foods (grapes), HC foods (muffins), and foods with an intermediate energy density (bread) were offered *ad libitum* 30 min following the preload. Energy intake was calculated to determine whether cognitive factors modulate subsequent food intake. We hypothesized that *ad libitum* food intake would be lower following the preloads that were presented with LC information and that incongruent calorie information would modulate the appetite hormone responses (ghrelin, insulin, and cortisol).

## Methods

### Participants

In a randomized, cross-over design with four conditions, 12 healthy young women [age: 23±1.8 y, BMI: 22.8±1.9 kg/m^2^, restraint score on the Three Factor Eating Questionnaire (22): 11±1.6], with regular breakfast intakes (equal to or more than five times a week), no regular intake of artificially sweetened products, and no use of sugar in coffee and/or tea, consumed a LC preload and a HC preload twice. Both preloads were provided once with LC information (LC-info) and once with HC information (HC-info). Exclusion criteria were as follows: hypersensitivity to the ingredients of the foods used in the study, reported lack of appetite, following an energy-restricted diet or a change in body weight of >5 kg during the last 3 months, or being a vegan/vegetarian. We explained the study procedures (described below) in a meeting preceding the experiment, in which participants also gave their written informed consent. Participants were, however, unaware of the exact aim of the study and were informed that we wanted to “investigate the body's response to the different energy loads”. They were asked to report the (guessed) aim after at the last session. All participants were debriefed after completion of their trials. The study was conducted according to the guidelines provided in the Declaration of Helsinki; all procedures were approved by the regional ethics committee of Uppsala (EPN), and the study is registered with http://ClinicalTrials.gov (NCT01680315).

### Design and procedures

**FIGURE 1:**
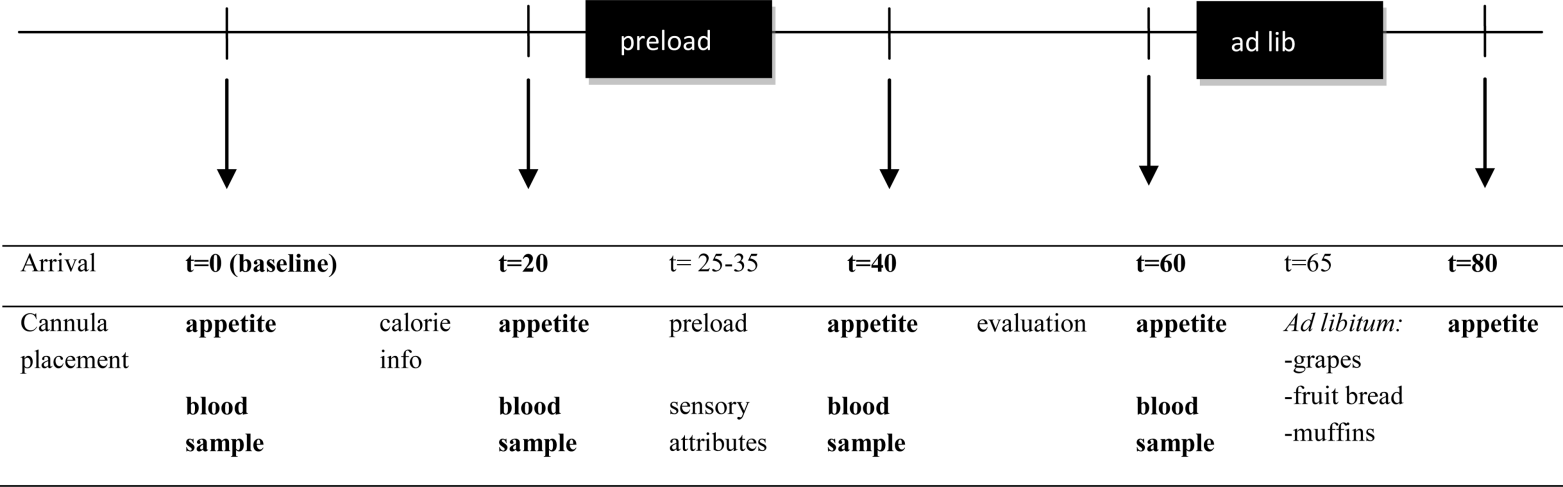
Experimental design. Blood was sampled every 20 min, and participants evaluated their appetite sensation (hunger, fullness, desire to eat, prospective consumption, and thirst) on a 100-mm visual analogue scale immediately after each blood sample.

Participants were instructed to refrain from alcohol, caffeine, food intake, and drinks except water after 22.00 the day before each test day and not to consume anything in the morning before arrival at the research centre at 07.30. Each session took place on a separate testing day, with a minimum wash-out period of 5 days between each session. Blood was sampled four times, at 0, 20, 40, and 60 min after inserting and using a venous cannula. Immediately after each blood sample, participants rated their appetite sensations (hunger, fullness, desire to eat, and prospective consumption) and thirst on 100-mm visual analogue scales (VAS), anchored “not at all” and “extremely”. Product information (described below) was provided after the first blood sample (*t* = 0-20), participants tasted, rated, and consumed the preload after the second blood sample (*t* = 20-40), and after the third blood sample (*t* = 40-60), participants filled out an evaluation questionnaire. Finally, they received their *ad libitum* breakfast after the fourth blood sample (*t* = 60-80). Following *ad libitum* consumption, participants once more evaluated their appetite sensation (*t* = 80; Figure 1). For tasting and rating the preloads, participants consumed one spoonful and evaluated the perceived pleasantness, sweetness, sourness, creaminess, and thickness of the yogurt on a 100-mm VAS, anchored “not at all” and “extremely”. This was repeated in the evaluation questionnaire. This procedure was repeated for all four conditions. The order of conditions was randomized within and balanced between participants.

### Test foods

Participants were served 300 g of LC yogurt (60 kcal/100 g) or HC yogurt (177 kcal/100 g) preloads. The HC preload consisted of 219 g of commercially available mild natural yogurt (*Mild lättyoghurt naturell*, Arla Foods), 66 g of table sugar, and 15 g of sunflower oil (Zeta). To match the LC preload in sweetness, 1.92 g artificial sweetener (cyclamate; *Suketter*: Cederroth International) dissolved in 8 g of water was added to 290 of the yogurt. The preloads were served in transparent cups.

After the preload, participants were provided with a breakfast plate that consisted of 300 g of white grapes (64 kcal/100 g), 10 triangles of fruit bread (∼18 g each) with 8 g of butter (312 kcal/100g; *Fruktkusar*; Fazer), and 5 muffins of 20 g each (430 kcal/100g; *Citronmuffins*; Hägges). Participants were provided an additional plate to avoid “empty your plate” behavior (23,24). This happened only twice, with the same participant, and both times when the provided product information stated LC content.

### Calorie information

In the “low-calorie information”-condition (LC-info), we provided a product description that stated that the preload is “a healthy breakfast product,” “light and fresh,” “low in fat and calories,” and “without added sugar”. We also provided a list of ingredients (including nonfat milk and vitamin A and D) and nutrition facts [energy (kcal) and macronutrients (g/100 g)]. In addition, participants were asked to calculate the energy percentage of protein, the total amount of calories in the product, and to describe the characteristics (picture, color, etc.) of a label and taste that would go best with the product. Every time the word “food” or “yogurt” was mentioned, it was preceded by the adjective LC. In the “high-calorie information”-condition (HC-info), the questionnaire was the same except for the product information (i.e., “a breakfast treat with the finest ingredients”, “that keeps away hunger flaws during the morning”, “with creamy yogurt cultured from whole milk”, and “rich product”), ingredient list (whole milk and milk proteins), nutrition facts, and the HC adjective.

When participants received the specific calorie information for the second time, they were asked to carefully consider the questionnaire. In addition to the previous visit with the same calorie information, participants also calculated the contribution of the preload to their daily energy requirements and compared the characteristics of the label (that they described) with food labels used for commercially available LC (HC) dairy products.

### Biochemical analysis

Blood samples were centrifuged directly after sampling, and the supernatant was stored at −80°C, for analysis of plasma glucose, insulin, cortisol, and ghrelin. Plasma glucose was measured using routine assays (hexokinase method, Aeroset; Abbott Diagnostics, North Chicago, IL). Noncompetitive immunometric assays were used to determine serum concentrations of insulin (12017547 122; Roche Diagnostics, Mannheim), and competitive assays were used to determine cortisol concentrations (11875116 122; Roche Diagnostics). Total ghrelin concentrations were assessed using commercially available ELISA kits for humans (EZGRT-89K; Millipore, Billerica, MA). Levels of total ghrelin were out of range for six samples of one participant. We therefore excluded all data from this participant for the ghrelin analyses.

### Data analysis

Continuous variables are presented as means (± SD). ANOVA (repeated measures) was used to test the effects of caloric content (content) and/or calorie information (info) on *ad libitum* energy intake, appetite ratings, blood parameters, and sensory attributes. Concentrations of blood parameters at *t* = 0 were included as a covariate, to adjust for differences across conditions. Tukey's post hoc tests were used to test for ANOVA-indicated differences, between the conditions. We included the time variable in the model to test for a time effect for the appetite ratings and blood parameters, and we tested for the effect of caloric content and caloric information on appetite sensations and blood parameters at the individual time points. Data were analyzed using SAS (version 9.3; SAS Institute). Results at a *P*-value of <0.05 were considered significantly different.

## Results

### Ad libitum energy intake

**FIGURE 2:**
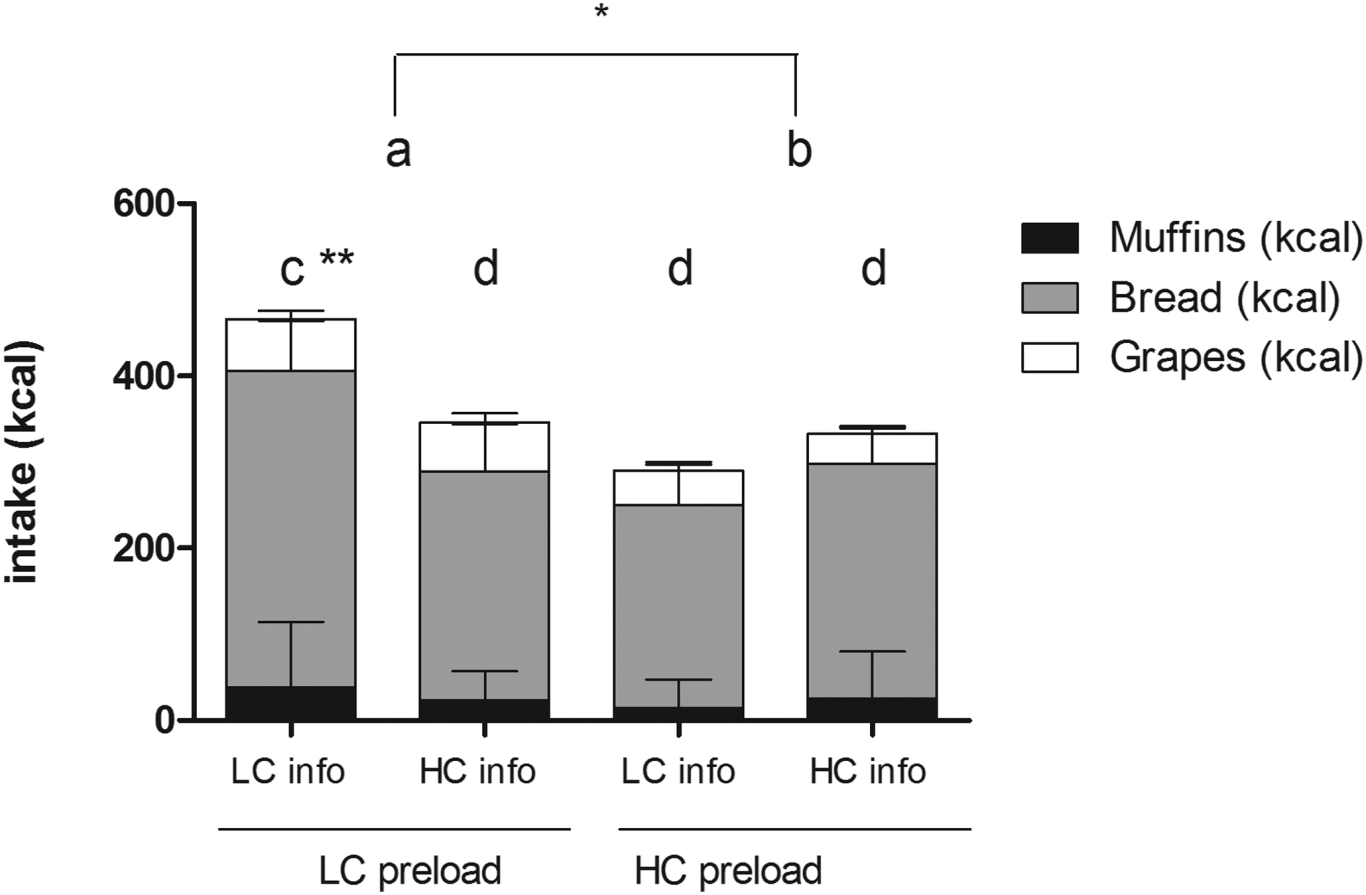
Mean intake (kcal ± SEM) of the muffins, fruit bread, and grapes product when offered *ad libitum* after the preloads that were either low-caloric (LC) or high-caloric (HC) and provided with the information that was either stating a low (LC-info) or a high (HC-info) caloric load. * The energy consumed from the breakfast plate was lower after the HC preloads^b^ as compared to the LC preloads^a^ (*P* < 0.01). ** Intake was lower after the LC-preload with the HC-info^d^ as compared to the LC-preload with the LC-info^c^ (content*info interaction: *P* = 0.03).

The amount of energy consumed from the breakfast plate depended on the caloric content of the preloads (*P* < 0.01) and on the information provided (content*info interaction: *P* = 0.03): *ad libitum* intake was higher when the LC preload was provided with the LC-info (Figure 2). Including BMI, body weight and/or restraint scores as covariates in the model did not change the results. Only two participants consumed at least one muffin. Repeating the analyses without the muffins, that is, including grapes and fruit buns only, did not change the result patterns.

### Blood parameters

**FIGURE 3:**
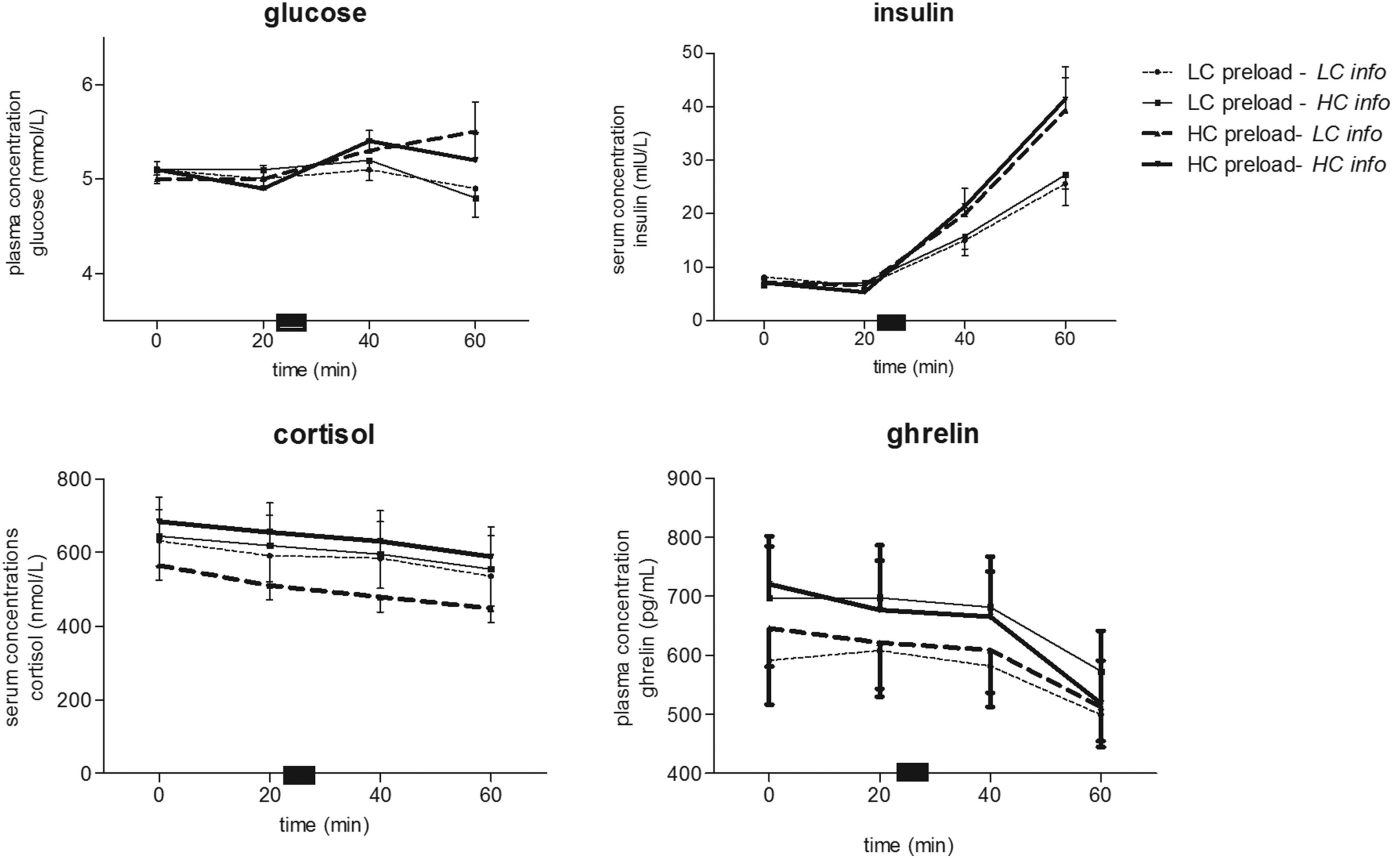
Mean (± SEM) values of glucose, insulin, cortisol, and total ghrelin for the low-caloric (LC) and high-caloric (HC) preloads, provided with the information that was either stating a low (LC-info) or a high (HC-info) calorie content. The small blocks on the *x*-axis represent consumption of the preload.

Baseline concentrations (*t* = 0) of glucose, insulin, and cortisol did not differ across conditions, but we observed higher ghrelin concentrations in the HC-info conditions at baseline (*P* = 0.03) (Figure 3). Both glucose and insulin levels depended on the caloric content (main effect content: glucose *P* = 0.01; insulin *P* < 0.001), with higher concentrations after consuming the HC preloads as compared to the LC preloads (*t* = 40 and *t* = 60). Cortisol concentrations depended on the provided caloric information (main effect: *P* = 0.05), but post hoc tests did not indicate significant differences between conditions, and cortisol concentrations did not differ across conditions at individual time points. Total ghrelin concentrations depended on the caloric content (main effect: *P* < 0.01), but post hoc tests did not show significant differences between conditions (Figure 3).

### Appetite ratings

**FIGURE 4:**
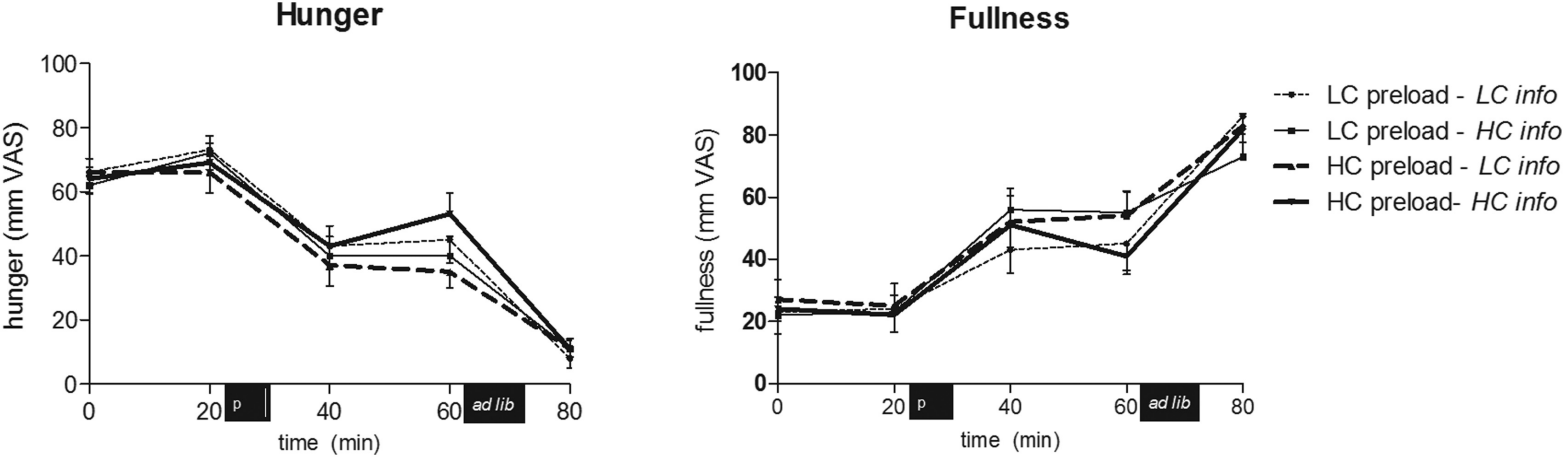
Mean (± SEM) values of hunger and fullness in conditions consuming the low-caloric (LC) and high-caloric (HC) preloads, provided with the information that was either stating a low (LC-info) or a high (HC-info) calorie content. The small blocks on the *x*-axis represent consumption of the preload (P) and the *ad libitum* test meal (ad lib).

As expected, hunger, desire to eat, and prospective consumption decreased, and fullness increased over time (all *P* < 0.0001). Ratings before (*t* = 0 and *t* = 20) and immediately after consuming the preload (*t* = 40) did not differ across conditions for any of the appetite sensations. At *t* = 60, we observed an interaction effect for both hunger and fullness (content*info: *P* < 0.01), while main effects of energy content and information were not significant. Post hoc tests showed that participants reported to be more hungry and after consuming the HC preload provided with the HC-info condition as compared to the HC preload with the LC-info (*P* =0.02) but did not indicate significant differences for fullness (Figure 4). Appetite sensations did not differ across conditions after *ad libitum* intake of items at the breakfast plate (*t* = 80).

### Sensory attributes and pleasantness

We did not observe differences in pleasantness ratings of the different preloads. The LC and HC preloads (independent of the information provided) were considered to be equally sweet and sour. Participants perceived the first bite of the LC preloads as more creamy (*P* = 0.09) and thicker (*P* = 0.06), but neither of these differences reached significance in the current sample (Table 1). However, ratings following consumption of the preload in its entirety showed the same pattern and reached significance (effect caloric content: creaminess *P* = 0.04; thickness *P* = 0.02). None of the participants guessed the exact aim of the experiment; three of them suggested that the aim was to study the effect of sweeteners and sugars on physiological responses.

**TABLE 1:** Ratings on pleasantness and sensory attributes (mean ± SD) for the low-caloric (LC) and high-caloric (HC) preloads (irrespective of the caloric information) and for the preloads provided with the LC-info and HC-info (irrespective of caloric content), as well as ratings of the four preloads

					LC foods		HC foods	
						
	LC foods	HC foods	Low info	High info	LC info	HC info	LC info	HC info
Pleasant	50 ± 27	44 ± 28	45 ± 29	49 ± 27	46 ± 30	54 ± 25	44 ± 29	45 ± 28
Sweet	84 ± 16	76 ± 21	82 ± 16	78 ± 21	83 ± 20	86 ± 11	82 ± 11	70 ± 26
Sour	19 ± 21	16 ± 16	17 ± 19	18 ± 18	22 ± 24	15 ± 17	11 ± 12	20 ± 19
Creamy	63 ± 24^a^	51 ± 24^b^	52 ± 25	61 ± 22	61 ± 24	65 ± 24	44 ± 25	58 ± 21
Thick	53 ± 19^a^	41 ± 23^b^	45 ± 22	50 ± 21	53 ± 20	54 ± 19	36 ± 21	47 ± 24

^b^LC preloads were perceived more creamy (*P* = 0.09) and thicker (*P* = 0.06).

## Discussion

The current study aimed to examine whether a mismatch between calorie content and calorie content label information of a preload determines subsequent *ad libitum* food intake and circulating appetite hormone levels (ghrelin, insulin, and cortisol) in healthy normal-weight women. To this aim, 12 female university students participated in four separate conditions: in two conditions, they consumed a LC preload (180 kcal), and in the other two conditions they consumed a HC preload (530 kcal). In addition, before consumption of each preload, they were provided with the calorie content information that was either equal or not equal to the actual calorie content of the preload. Independent of the calorie content information, *ad libitum* food intake was significantly lower when they were administered a HC preload. When assuming that the LC preload would be dense in energy, subsequent *ad libitum* food intake was lower than that following the LC preload with the consistent calorie content information. Such an effect was not observed between the HC preload conditions. Finally, the hormonal response to the preload was driven by its energy content, rather than by its information. These results suggest that the effect of recent eating on subsequent food intake is moderated by an interaction of calorie content and personal caloric expectations, whereas hormonal changes induced by food intake are not sensitive to psychological bias.

The observed effect of calorie anticipation on subsequent food intake is in line with previous findings. For instance, in a previous study, female participants were exposed to a “lunch cue” (in which they were asked to think about what they had eaten for lunch) or “no cue” (free thought condition) for 5 min prior to eating. Participants ate less following exposure to the “lunch cue” than the “no cue” condition (25). It has also been observed that imagining eating a food alone (i.e., without actual consumption) can modulate subsequent food intake (26). This suggests that retrieval of memory for recent eating plays a major role for subsequent food intake (27). Going beyond these findings, we demonstrated that encoding of the information that a preload is energy dense represents a sufficient stimulus to reduce subsequent *ad libitum* food intake in young female adults. In line with our findings, previous studies have demonstrated that having the belief that one has eaten a considerable amount of calories influences fullness and feelings of satiety (28,29). However, at this point, it is important to emphasize that the inconsistent calorie content information only affected subsequent food intake in the LC preload conditions, that is, no such effects were seen for the HC conditions. One explanation for these discrepant results might be that once the preload has supplied a certain number of calories, cognitive processes related to food intake play an inferior role for subsequent eating.

There are some mechanisms through which the inconsistent calorie content information in the LC conditions may have reduced subsequent *ad libitum* food intake in our study. For instance, assuming that the preload was dense in energy may have resulted in increased activation of neural circuits involved in suppression of hunger and food intake, such as the dorsolateral prefrontal cortex (30). It has also been observed that labeling food as low fat can increase intake of a snack and reduce associated guilt, especially in overweight subjects (31), and that anticipation of a low-calorie milkshake results in less brain activation in reward-related areas in obese relative to lean women (32).

While the calorie content information affected subsequent food intake in LC conditions, no such an effect was observed for the endocrine response, including ghrelin, cortisol, and insulin. At first glance, this result appears to be in contrast to previous findings in which meal anticipation yielded an increase in concentrations of serum cortisol concentrations (18) or in plasma concentrations of ghrelin (11). However, this discrepancy is most likely caused by differences in study settings: meal anticipation compared to no meal anticipation (18) versus food anticipation in all conditions, as well as differences in the studied time period (11). Limitations in our study may also have led to discrepancies from prior results. First, we did not select participants based on a restrained eating behavior score. A high dietary restraint score refers to the tendency to control food intake at a cognitive level (33). Including only restrained eaters may have resulted in a more pronounced effect of the calorie information on food intake. Second, we had a relatively low blood sampling frequency, which may have masked possible alterations of the measured hormones. Finally, we only studied normal-weight healthy females and generalization to males or other weight abnormalities is not appropriate. In summary, our results suggest that both metabolic and cognitive features of food affect subsequent food intake decisions in healthy young women. Bearing the small sample size in mind, additional studies are needed to elucidate further the effects and underlying mechanisms through which cognitive cues related to food intake affect subsequent eating behavior in humans.
